# The role of putative human anterior intraparietal sulcus area in observed manipulative action discrimination

**DOI:** 10.1002/brb3.1226

**Published:** 2019-02-11

**Authors:** Guy A. Orban, Stefania Ferri, Artem Platonov

**Affiliations:** ^1^ Department of Medicine and Surgery University of Parma Parma Italy

**Keywords:** featural attention, fMRI, posterior parietal cortex, selective neurons, two‐alternative forced choice

## Abstract

**Introduction:**

Although it has become widely accepted that the action observation network (AON) includes three levels (occipito‐temporal, parietal and premotor), little is known concerning the specific role of these levels within perceptual tasks probing action observation. Recent single cell studies suggest that the parietal level carries the information required to discriminate between two‐alternative observed actions, but do not exclude possible contributions from the other two levels.

**Methods:**

Two functional magnetic resonance imaging experiments used a task‐based attentional modulation paradigm in which subjects viewed videos of an actor performing a manipulative action on a coloured object, and discriminated between either two observed manipulative actions, two actors or two colours.

**Results:**

Both experiments demonstrated that relative to actor and colour discrimination, discrimination between observed manipulative actions involved the putative human anterior intraparietal sulcus (phAIP) area in parietal cortex. In one experiment, where the observed actions also differed with regard to effectors, premotor cortex was also specifically recruited.

**Conclusions:**

Our results highlight the primary role of parietal cortex in discriminating between two‐alternative observed manipulative actions, consistent with the view that this level plays a major role in representing the identity of an observed action.

## 1 INTRODUCTION

Recent single cell studies, published in abstract form (Lanzillotto et al., 2019; Orban et al., 2016), have demonstrated that monkey anterior intraparietal area (AIP), as well as its putative human homologue (phAIP, Orban, 2016) hosts sizeable proportions of neurons selective for observed manipulative actions (OMAs), extending an earlier AIP study (Pani, Theys, Romero, & Janssen, 2014). OMA‐selective neurons responded strongly to one of the seven OMAs tested but less so to the other six. Furthermore, in both species a population of a hundred neurons, having random degrees of selectivity, were able to decode the seven OMAs with accuracies exceeding 80%. Humans are able to discriminate between pairs of OMAs even in the presence of noise and for short presentations of the actions (Platonov & Orban, 2016). This invites a straight forward prediction that can be tested with functional imaging: if humans, when performing this task, indeed rely on the information provided by these neurons, then phAIP should be active in comparison to a control task not involving judgments about OMAs.

Functional imaging studies, however, have established (Caspers, Zilles, Laird, & Eickhoff, 2010; Cross, Kraemer, Hamilton, Kelley, & Grafton, 2009; Jastorff, Begliomini, Fabbri‐Destro, Rizzolatti, & Ga, 2010) that observation of manipulative actions activates a network including posterior middle temporal gyrus (pMTG) and posterior occipito‐temporal sulcus (pOTS) at the occipito‐temporal level and the precentral (PCS) region at the premotor level, in addition to phAIP at the parietal level. Studies in the monkey in which functional imaging was combined with anatomical evidence (Nelissen et al., 2011) indicate that this action observation network (AON) is hierarchically organized, with premotor being the higher level and occipito‐temporal the lower level. While little or no information is available regarding OMA selectivity at the occipito‐temporal or premotor levels, older single cell studies have indicated that neurons at these levels are also responsive to the observed actions of others (Gallese, Fadiga, Fogassi, & Rizzolatti, 1996; Ferrari et al., 2003, Perrett et al., 1989; Singer & Sheinberg, 2010; Vangeneugden, Pollick, & Vogels, 2009). Thus we cannot exclude the possibility that the other two levels of the AON may also be engaged by discrimination between OMAs.

To distinguish between these two alternatives, we used a task‐based attentional modulation paradigm manipulating featural attention to a constant stimulus (Cant & Goodale, 2007; Chiu, Esterman, Han, Rosen, & Yantis, 2011; Peuskens et al., 2004), comparing tasks in which subjects viewed identical video clips and made judgments either about the action portrayed, the colour of the object manipulated, or the gender of the actor. This type of experiment does not test whether phAIP has the capability to discriminate between OMA exemplars, as an multi voxel pattern analysis (MVPA) experiment in attentive subjects would do (Oosterhof, Tipper, & Downing, 2012; Wurm & Lingnau, 2015). Indeed, the single neuron studies previously referenced have already provided a much stronger demonstration. It can, however, directly demonstrate the selective recruitment of phAIP when subjects actually perform the OMA discrimination. The first functional magnetic resonance imaging (fMRI) experiment tested the discrimination of two manipulative actions performed with the right hand, as in the psychophysical studies (Platonov & Orban, 2016). To demonstrate the generality of our results, we introduced an additional difference in the discriminanda in a second fMRI study, comparing two manipulative actions performed with either one or two hands. As the bimanual and unimanual actions differ even in the static frames at action onset, we performed a psychophysical control experiment to verify that subjects did not use static cues in discriminating between OMAs.

## 2 MATERIALS AND METHODS

### 2.1 Participants

Twenty‐eight and 22 right‐handed, healthy volunteers with normal or corrected‐to‐normal visual acuity, who had successfully mastered the discrimination tasks, participated in the first and second fMRI experiment respectively. From these, four and three subjects were eliminated because of excessive head motion, leaving 24 (12 females; mean age: 23.6 years, range: 20–30) and 19 subjects (10 females; mean age: 27.4 years, range: 22–32) participating in the first and second experiments respectively We selected the sample size based on the Desmond and Glover (2002) study according to which 20–24 subjects are required for retaining 80% of the power at the single‐voxel level using conservative thresholds. Subjects were naïve as to the purpose of the experiment and gave informed consent for participation. Experiments were carried out according to the national and European guidelines for testing human subjects, and all experimental protocols were approved by the Ethics committee of the Province of Parma.

### 2.2 Stimuli and design

**Figure 1:**
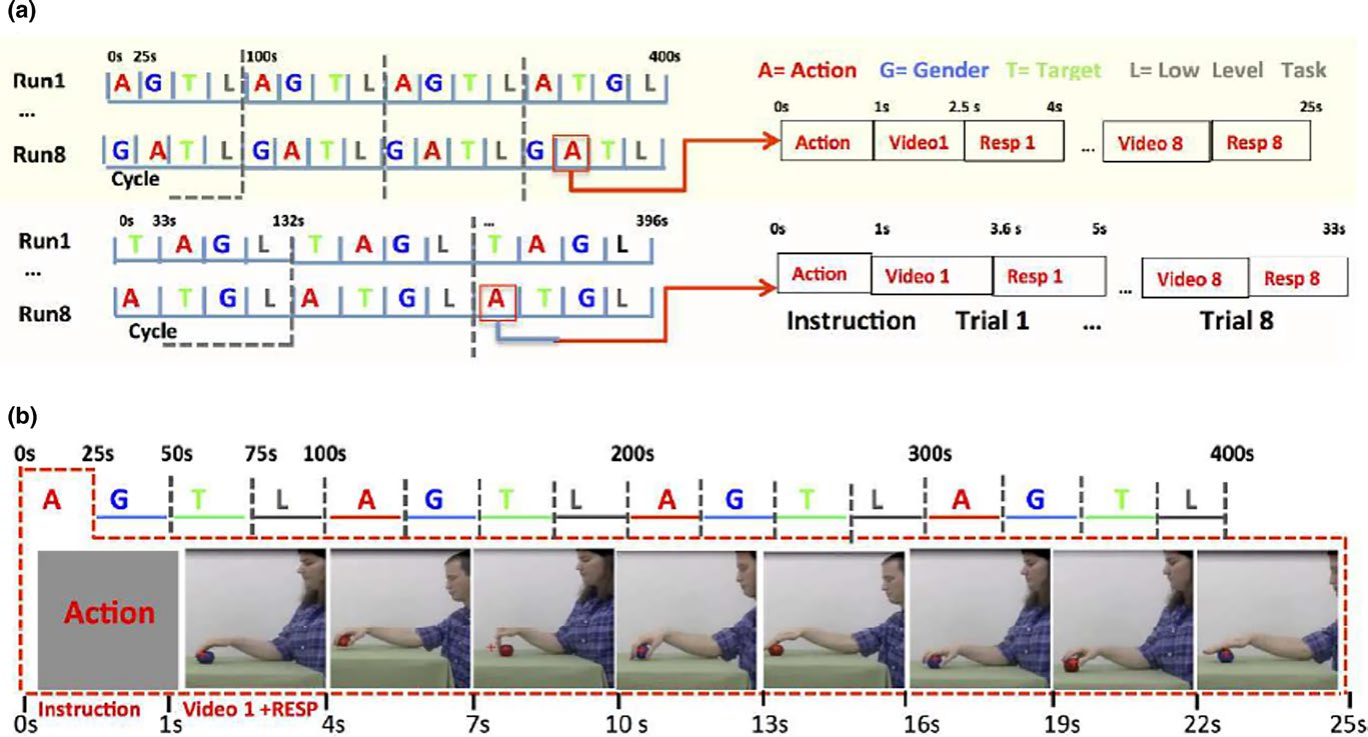
Description of the fMRI experiments: (a) run and block structures in experiments 1 (top) and 2 (bottom); (b) randomization of main factors (action exemplars, gender of actor and colour of target) in a block. In (a), videos lasted longer in experiment 2 than in experiment 1, hence differences in block and run structures

The experimental stimuli consisted of videoclips showing an actor standing at the right or the left of a table and manipulating a coloured object lying on the table (Figure 1). Video edges were blurred with an elliptical mask (14^0^ × 10^0^), whereby the videoclip gradually melded into the black background, avoiding a sharply contrasting border between video and background. The videoclips were common to all three experimental conditions, which were defined by the three features of the videos on which subject based their judgements: observed manipulative action, gender of the actor, and colour of the object (Figure 1a). In the discrimination trials, the videoclips were followed by the presentation of a blank screen of average luminance to allow the subject time to respond. All three experiments used an explicit baseline task, in which a mean luminance screen was shown for the same duration as the discrimination trials. The subjects performed a 2AFC discrimination task when viewing videoclips, indicating their response by pressing one of two buttons with either the index or middle finger of the right hand. All subjects used the same fingers but these were assigned opposite functions in each half of the subjects. Subjects discriminated either action (dragging, grasping or grasping, pushing), gender of the actor (male or female), or colour of the object (blue, red), while making random choices in the baseline task. The subjects were instructed to watch the movie, to hold fixation on a small coloured square presented near the target object or the body part, and to give their response when ready. Data were collected in a single 8‐run session in the three experiments.

The stimuli for experiment 1, modified from Platonov and Orban (2016), consisted of 1.5 s videoclips showing a standing actor dragging or grasping an object lying on a table using only the right hand. The two manipulative actions could be performed by a male or female actor on a blue or red ball; moreover the starting point of the hand could be positioned either above or on the table. Including the two positions of the actor in the visual field, this generated 2^5^ = 32 videos, 16 per OMA exemplar (Table 1). The red fixation point was placed in the middle of action trajectory. Each block, corresponding to a single condition, included: 1 s panel with the instruction in Italian (see Figure 1b), and eight trials including 1.5 s for videoclips and 1.5 s response periods. Thus each block lasted 25 s, and followed each other without gap. The total duration of a run, in which the four conditions (action, gender, colour and baseline) were presented four times, was 400 s (Figure 1).

**Table 1:** Factors randomized in the designs of the two experiments

Factor	Action	Gender	Target	Actor position	Starting position
Experiment					
Exp 1	Grasping/dragging	Male/female	Red/blue	Left/right	Above/on
Exp 2	Grasping/pushing	Male/female	Red/blue	Left/right	

Experiment 2 utilized 2.6 s videoclips taken from Ferri, Rizzolatti, and Orban (2015), showing a standing actor pushing an object lying on the table with two hands or grasping it with one hand. The two manipulative actions could be performed by a male or female actor on a small red cube or a large blue ball. Including actor position, this generated 16 videos, eight per exemplar (Table 1). The yellow fixation point was displaced up (in four runs), or down (remaining four runs) to the object‐hand pair. Each block‐condition included: a 1 s panel with the instruction in Italian (translated into English in the figure), and eight trials including a 2.6 s of videoclips followed by 1.4 s response period. Each block lasted 33 s, yielding 396 s runs in which the four conditions were presented three times (Figure 1a).

In both experiments, the eight videos generated by the three factors to be discriminated were presented in random orders within a block (Table 1, Figure 1), with the block order of the pseudo‐randomly selected within a cycle (Figure 1b), and counterbalanced across runs and participants. Other factors were accommodated in the design (Table 1): position of the actor in visual field halves in alternating runs and the factor hand position in alternating cycles of a run. In both experiments, eight runs were collected in a single session.

### 2.3 Psychophysical control experiment

The videos used in fMRI experiment 2 showed either unimanual or bimanual OMAs. As this difference in the effector was present from the first frame onwards, subjects could arguably use this static cue to make their judgments. Hence, in a control experiment, we examined the perception of the first frame of the videos, while subjects (10, four females, mean age 26.7 years, range: 25–29) were discriminating the observed actions, just as they did in the scanner.

#### 2.3.1 Set‐up

Subjects were seated 72 cm from a liquid crystal display (Samsung, T27A950, resolution 1,920 × 1,080 pixels, 50 Hz refresh rate) in an otherwise dark room with their heads supported by a forehead rest and a chin cup. Subjects were instructed to fixate a small target in the centre of the screen. Eye position was recorded in all but one subject, using a noninvasive monitor‐mounted infrared video system (Tobii Version X2‐60) sampling the positions of both eyes at 60 Hz.

#### 2.3.2 Task and stimuli

**Figure 2:**
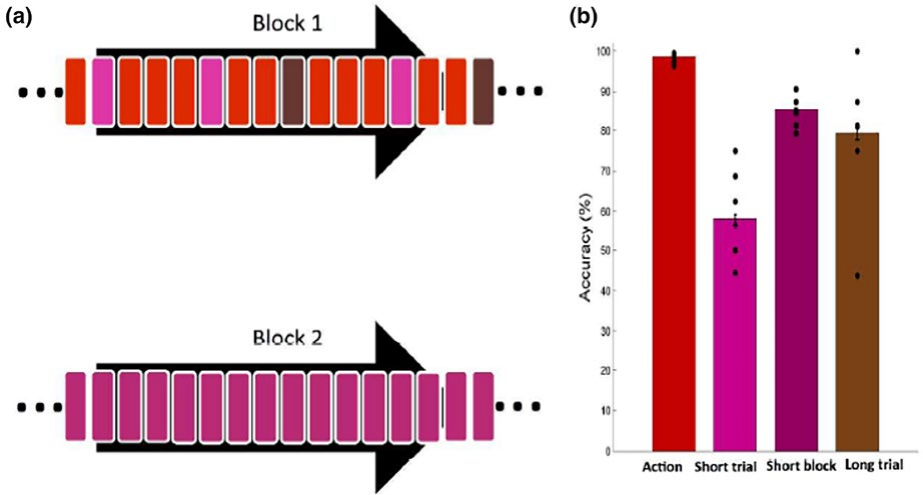
Psychophysical static control experiment. (a) Example of the block structure. In Block 1, action videos (red rectangles) were pseudo‐randomly replaced by both short static‐probe stimuli (light purple rectangles) and long static‐probe stimuli (brown rectangles), such that one static stimulus could not immediately follow another. Block 2 consisted of short static stimuli only (dark purple rectangles). Two blocks were presented randomly in one session. (b) Mean accuracy for discriminating action videos (red bar), short static‐probe trials (light purple bar), short static blocks (dark purple bar) and long static‐probe trials (brown bar). Black dots: individual subjects (*n* = 10); vertical lines *SE*

We used a two‐alternative forced‐choice (2AFC) discrimination task in which subjects viewed a single video and indicated their choices (actor grasping an object with one hand and pushing it with both) by pressing one of two buttons with the right hand, as soon as they were ready. We created eight videos (17° × 13°, 50 Hz) for each action exemplar by combining two actors (male, female) × 2 objects (bigger ball, smaller cube) × 2 lateral viewpoints (left, right in the visual field). In addition, two other types of stimuli were produced. In the short and long static stimuli, the first frame of each version of the action movies was displayed for durations of 40 or 200 ms (static phase), whereas the remaining of the 2.6 s stimuli was made of 0% signal level dynamic noise. In the control blocks, we presented the short static stimuli separately, while in the test blocks the static‐probe stimuli were interspersed amongst the video presentations (Figure 2a).

The 256 (eight versions of two action exemplar videos, repeated 14 times each) action movies were presented in random order in a single test block (Figure 2a, block 1, red). In addition to these, the same block also contained 16 (8 × 2) short static‐probe trials (Figure 2a, block 1, light purple) and 16 (8 × 2) long static‐probe trials (Figure 2a, block 1, brown) presented pseudo‐randomly such that two static trials did not follow each other. The other, short‐static block (Figure 2a, block 2) consisted of 32 short static trials (16 trials repeated twice, dark purple). The two blocks were presented in random order to each subject in a single session.

#### 2.3.3 Results

Subjects fixated well in this control experiment, with the standard deviation of eye position being similar in both the test (averages 0.90° and 0.90° for horizontal and vertical directions) and control (averages 0.94° and 0.67° for horizontal and vertical directions) blocks. Their accuracies for the videos were near 100% correct (98.6 ± 0.12), significantly higher than the accuracy in any static condition (paired two‐sided *t* test, *t*_9_ = 12.4, *p* < 0.01; *t*_9_ = 10.1, *p* < 0.01; *t*_9_ = 3.97, *p* < 0.05, for short static trials, short static blocks and long static trials respectively). Accuracy in short static‐probe trials was near chance (58.2 ± 1.0), but significantly increased in long static‐probe trials (79.3 ± 1.64, *t*_9_ = 4.65, *p* < 0.01). Interestingly, in the control blocks, accuracy was not different from that in long static‐probe trials (85.4 ± 0.44, *t*_9_ = 1.13, *p* > 0.05) and significantly better than for short static probe presentations (*t*_9_ = 7.12, *p* < 0.01). Thus subjects viewing videos to discriminate action exemplars were unable to use brief static frames to form judgments, even though those frames were discriminable when shown on their own. This deficit was observed only for short static probes, as these subjects could discriminate longer static‐probe stimuli, ruling out simple attention effects. This control experiment strongly suggests that static cues were not used in the action discrimination of fMRI experiment 2, even if momentarily present at the start of the video presentation.

### 2.4 Data collection

Before the scanning session, all observers were trained to discriminate between the action/gender/colour alternatives. The structure of the training session was similar to that described for the fMRI session except for the absence of low‐level task blocks, and auditory feedback provided after the response. The procedure was repeated until subjects made <1 error per 50 trials. Three and two subjects in experiments 1 and 2 respectively were unable to perform the task correctly after five familiarization blocks were not included in the fMRI experiment, leaving 28 and 22 participants in the first and second experiment respectively.

Visual stimuli were presented in the fronto‐parallel plane by means of a head‐mounted display (60 Hz refresh rate) with a resolution of 800 × 600 pixels (Resonance Technology, Northridge, CA) in each eye. The display was controlled by an ATI Radeon 2400 DX dual output video card (AMD, Sun Valley, CA), driven by E‐Prime software (Psychology Software Tools, Sharpsburg, PA). To reduce head motion, the subjects’ head was restrained with cushions. Subjects indicated responses by pressing a button under the index or middle finger using a response box (Resonance Technology, Northridge, CA) positioned under the right hand. Throughout the scanning session, eye movements were recorded with an infrared eye tracking system (60 Hz, Resonance technology, Northridge, CA). Scanning was performed using a 3T MR scanner (GE Discovery MR750, Milwaukee, ILL) with an 8‐parallel‐channels receiver coil, in the Hospital of Parma. Functional images were acquired using gradient‐echoplanar imaging with the following parameters: 49 horizontal slices (2.5 mm slice thick‐ ness; 0.25 mm gap), repetition time (TR) = 3 s, time of echo (TE) = 30 ms, flip angle = 90°, 96 × 96 matrix with FOV 240, and ASSET = 2, 1,212 and 1,089 volumes collected for experiments 1 and 2 respectively, 49 slices covering the entire brain in each volume. A 3D T1‐weighted IR‐prepared fast SPGR (Bravo) image was acquired and used for anatomical reference with these parameters: TE/TR 3.7/9.2 ms; inversion time = 650 ms, flip angle = 128, ARC = 2; 186 sagittal slices acquired with 1 × 1 × 1 mm^3^ resolution.

### 2.5 Data analysis

Preprocessing (SPM8 software, Welcome Department of Cognitive Neurology, London, UK) involved: (a) realignment, (b) co‐registration of anatomical and mean functional images, (c) spatial normalization to standard MNI152 space (by estimating the optimum 12‐parameter affine transformation and nonlinear deformation with voxel size of 2 × 2 × 2 mm) and (d) smoothing (isotropic Gaussian kernel of 6 mm). For four subjects in experiment 1, 2 runs were discarded because more than 10% of the volumes were corrupted, (scan to scan movement exceeded 0.5 mm per TR in any of the six realignment parameters according to art repair in SPM8). Two corrupted runs, corresponding to retaining 75% of the data collected, were designated as the threshold for excluding runs while still keeping the subject in the analysis.

We next applied to the eight runs a GLM composed of 10 regressors: four for conditions plus six motion regressors. The condition‐specific regressors were convolved with the canonical hemodynamic response function (HDR). In experiments 1 and 2, three simple contrasts were defined at the subjects’ level: 1. Action versus actor discrimination, 2. Action versus colour discrimination and 3. Action discrimination versus low‐level task. Both the specific and general maps were generated at the second, random‐effects level. The specific map was defined by the conjunction (conjunction null, Nichols, Brett, Andersson, Wager, & Poline, 2005) of the contrasts comparing the action discrimination condition to the other two discrimination conditions, inclusively masked with the contrast task condition versus low‐level task at *p* < 0.01 uncorrected level. This map was thresholded at *p* < 0.05 family‐wise error (FWE) corrected (based on random field theory), corresponding to a *t* score of 5.6. For visualization purposes, a lower threshold of *p* < 0.001 uncorrected was used. The general map was defined by the contrast comparing the action discrimination task with the low‐level, active fixation condition, simply thresholded at *p* < 0.001 uncorrected level.

**Figure 3:**
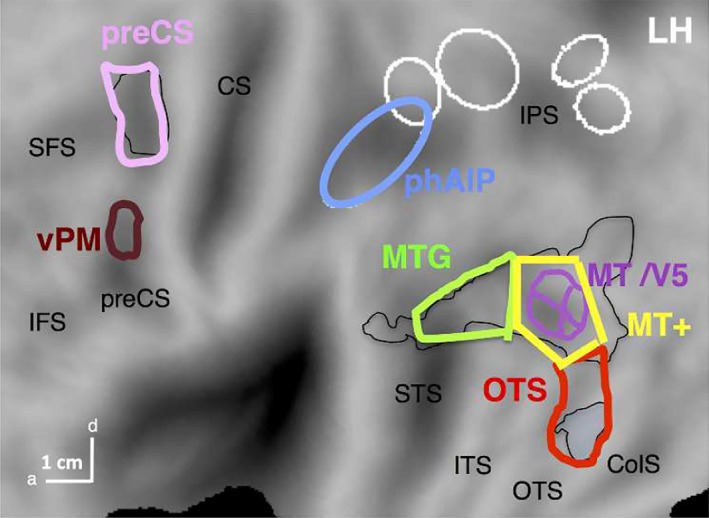
A priori ROIs created from the Ferri et al. (2015) left activation map (black line). Lateral occipito‐temporal (LOTC), and the premotor clusters of left activation map, thresholded at *p* < 0.01, were plotted in Caret (black lines) and subsequently subdivided and adapted following both anatomic and functional criteria. The MTG ROI (light green) was defined as the MTG part of the LOTC cluster including the extension into the STS; the OTS ROI (red) covered the posterior part of OTS sulcus, extending from the rostral boundary of the overall LOTC cluster, to the rostral boundary of the OTS cluster; the MT + ROI (yellow) was located between the MTG and OTS ROIs and surrounded the retinotopic MT cluster (violet). Finally the main dorsal preCS ROI (pink) was created keeping only the activation occupying precentral sulcus, to avoid supplementary motor and primary motor regions respectively. The number of voxels per ROI was: 449, 470, 417 and 328 for MT+, OTS, MTG, and preCS ROIs respectively. We identified the voxel coordinates contained in each ROI, exported into MNI space and created the right counterpart, when needed. Both left and right phAIP (blue) ellipses (Jastorff et al., 2010) were also included, with 419 and 323 voxels respectively (other ellipses see Figure 4). Finally, a ventral premotor ROI (vPM, brown) was defined from a recent study of observing manipulation actions with no reaching component (Corbo et al., 2016)

The ROI analysis used a priori ROIs (Figure 3) obtained from the left hemisphere activation map for observing manipulation in Ferri et al. (2015). These occipito‐temporal (MTG, OTS) and premotor (preCS) ROIs were thus derived from the same study as the stimuli used in experiment 2, which also included the exemplars grasping and dragging used in experiment 1. Activation of these regions was observed in many of our previous experiments (Abdollahi, Jastorff, & Orban, 2013; Corbo & Orban, 2017; Jastorff et al., 2010), using similar experimental and control stimuli to those used in the present study. Hence these ROIs were more appropriate than those derived from any meta‐analyses (e.g. Caspers et al., 2010) averaging over studies with often very different experimental and control stimuli from those used here. The MTG and OTS regions overlap largely with the lateral occipito‐temporal cortex, implicated in action observation (Lingnau & Downing, 2015), except for the omission of the caudal part, corresponding to the MT + ROI of Ferri et al. (2015). We did not include this caudal region as it corresponds broadly to the MT cluster (Kolster, Peeters, & Orban, 2010), of which the homologous areas in the monkey are known to process low‐level motion parameters. At the parietal level, we used the phAIP ellipse (Jastorff et al., 2010), which was also included in the activation map of Ferri et al. (2015). At the premotor level, we considered a more ventral ROI (vPM) from an unpublished study (Corbo, Aflalo, Andersen, & Orban, 2016), but this ROI proved inactive and will not be analysed further. The activity profiles (split analysis) were tested statistically with three‐way ANOVAs, complemented by one‐way ANOVAs (with Bonferroni correction).

All maps were projected onto the flattened left and right hemispheres of the human PALS B12 atlas [(Van Essen, 2005) http://sumsdb.wustl.edu:8081/sums/ directory.do?id=636032] using the Caret software package [(Van Essen, Drury, Dickson, Harwell, & Anderson, 2001), http://brainvis.wustl.edu/caret]. Other relevant ROIs are indicated on these flat maps: pink and green outlines correspond to cytoarchitectonically defined areas in parietal operculum (Eickhoff, Jbabdi, Caspers, Laird, & Zilles, 2007) and inferior parietal lobule (IPL) regions (Caspers et al., 2006) respectively; white ladder‐like outline: premotor mini‐ROIs (Jastorff et al., 2010). Gold and black outlines: maximum probability maps (MPM) of V1‐3 (gold) and retinotopic regions beyond early visual cortex (black, Abdollahi et al., 2014).

## 3 RESULTS

### 3.1 fMRI Experiment 1

In this fMRI study, subjects watched videos showing an actor grasping or dragging an object (Table 1), and performed one of three 2‐AFC tasks regarding the observed action, gender of the agent or colour of the object (Figure 2). Twenty‐four right‐handed subjects were successfully scanned in a single fMRI session. They fixated well during scanning (average: 8.2 saccades per min, *SD* = 1.7), with no significant differences across conditions (ANOVA *F*_3,18_ = 0.7, *p* > 0.8). Steady fixation was essential, as several cortical regions including parietal cortex display BOLD responses highly correlated with saccade frequency (Kimmig et al., 2001). The mean accuracy of the responses was high in all three 2‐AFC tasks: action = 96.3% (*SD* 1.3%), actor = 97.1% (*SD* 1.5%), colour = 96.6% (*SD* 1.7), indicating that the subjects attended to the stimuli and understood the instructions well. Mean reaction times were much longer for action discrimination (0.94 s, *SD* 0.24 s) than for actor (0.49 s, *SD* 0.19 s) or colour (0.31 s, *SD* 0.17) discrimination.

**Figure 4:**
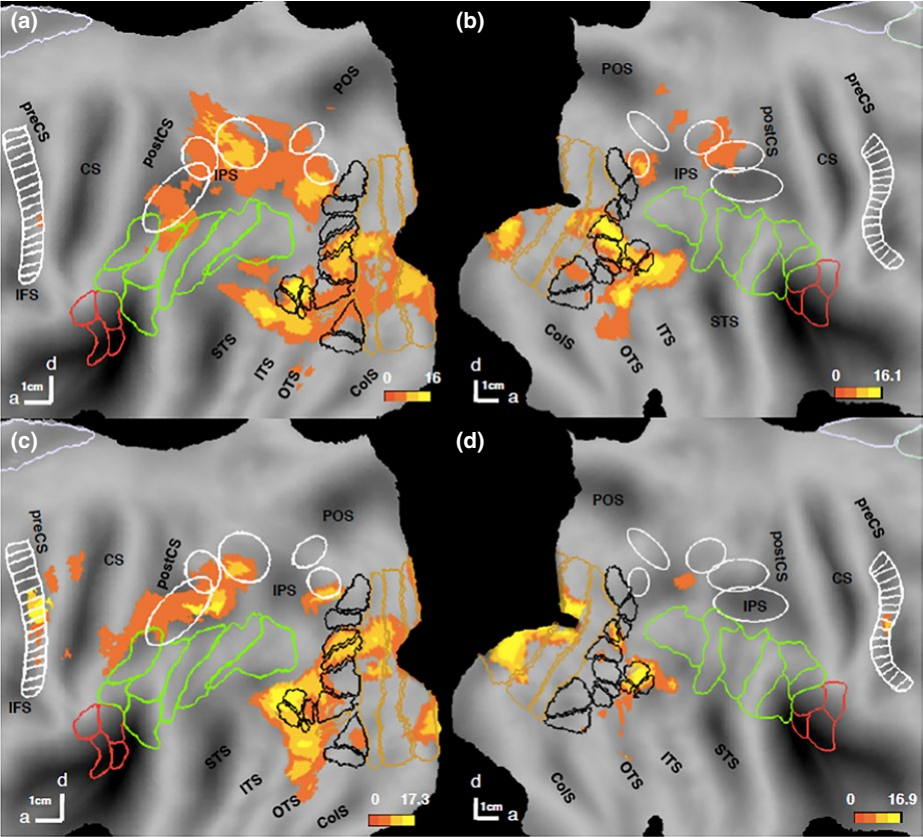
Regions involved in observed action discrimination (general map): statistical parametric maps (SPMs) showing the significant sites for action discrimination versus active fixation in experiment 1 (a, b) and experiment 2 (c, d) on the flattened left (a, c) and right (b, d) hemispheres (posterior parts). Pink and green outlines correspond to cytoarchitectonic areas in opercular (Eickhoff et al., 2007), and IPL (Caspers et al., 2006) regions respectively. White ellipses are confidence ellipses for phAIP, DIPSA, DIPSM, POIPS, and VIPS (from rostral to caudal) and white ladder‐like outlines are premotor mini‐ROIs (Jastorff et al., 2010). Black outlines: maximum probability maps (MPMs) of retinotopic regions beyond EVC; gold outlines: MPMs of V1–V3 (Abdollahi et al., 2014). preCS, precentral sulcus; CS, central sulcus; postCS, postcentral sulcus; IPS, intraparietal sulcus; STS, superior temporal sulcus; ITS, inferior temporal sulcus; OTS, occipito‐temporal sulcus; ColS, collatereral sulcus

When compared to the active fixation baseline (Figure 4), action discrimination activated retinotopic regions (mainly early visual cortex V1‐V3, the MT cluster and LO1/V3A), and the two lower levels of the AON. The latter activation was stronger in the left hemisphere than the right, even if the main source for such asymmetry, the position of the actor in the visual field had been removed. The occipito‐temporal activations included pMTG and pOTS, consistent with many other studies (Abdollahi et al., 2013; Ferri et al., 2015; Jastorff et al., 2010) and at the parietal level caudally regions close to ventral intraparietal sulcus (VIPS) and more rostrally, dorsal intraparietal sulcus medial/anterior (DIPSM/DIPSA) regions.

**Figure 5:**
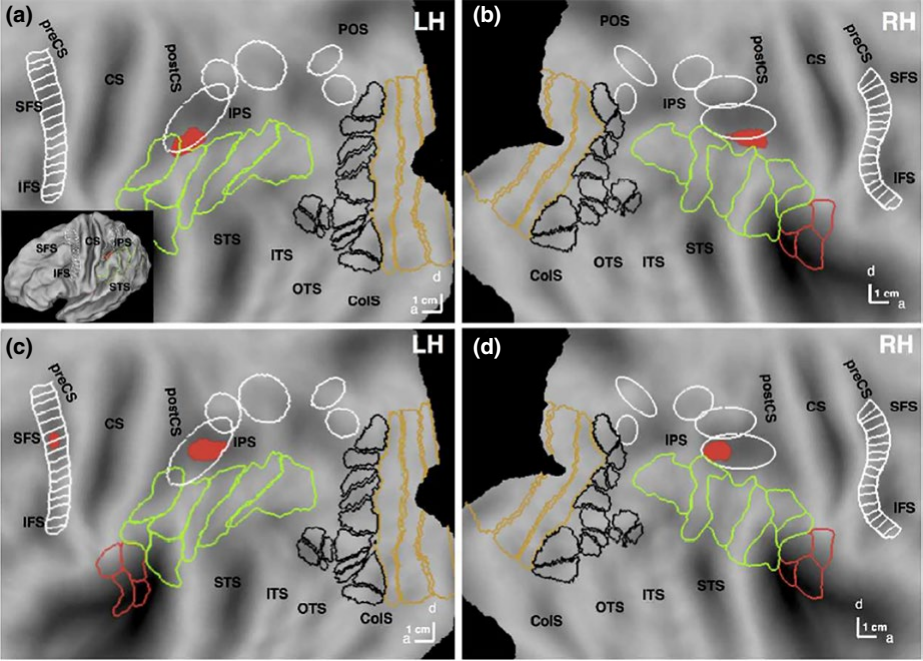
Regions specifically involved in observed action discrimination: statistical parametric maps (SPMs) showing the significant (local maxima FWE corrected *p* < 0.05) sites (Table 2) for conjunction of action (red) discrimination versus the two other discriminations in experiment 1 (a, b) and experiment 2 (c, d) on the flattened left (a, c) and right (b, d) hemispheres (posterior parts). Inset in a: lateral view of folded hemisphere. White ellipses (from rostral to caudal) are confidence ellipses for putative human Anterior intraparietal (phAIP), dorsal intraparietal sulcus anterior (DIPSA), dorsal intraparietal sulcus medial (DIPSM), parieto‐occipital intraparietal sulcus (POIPS) and ventral intraparietal sulcus (VIPS) (from rostral to caudal). White ladder‐like outline: premotor mini‐ROIs (see methods). Pink and green outlines correspond to cytoarchitectonically defined areas in parietal operculum and inferior parietal lobule (IPL) regions respectively (see methods). Black outlines: maximum probability maps (MPM) of retinotopic regions beyond early visual cortex; gold outlines: MPMs of V1–V3 (see methods). SFS, superior frontal sulcus; IFS, inferior frontal sulcus; preCS, precentral sulcus; CS, central sulcus; postCS, postcentral sulcus; IPS, intraparietal sulcus; POS, parieto‐occipital sulcus; STS, superior temporal sulcus; ITS, inferior temporal sulcus; OTS, occipito‐temporal sulcus; ColS, collatereral sulcus

To map the regions *selective* for each discrimination task, we performed a conjunction of the contrasts comparing this discrimination condition with the other two (actor and colour) in a whole‐brain analysis. Only two cortical regions (Figure 5a,b, Table 2) were more active when subjects discriminated actions rather than either of the two other features: phAIP bilaterally (left: −50 −38 44, *z* = 5.7, *p* < 0.05 FWE corrected; right: 42 −38 48, *z* = 5.7, *p* < 0.05 FWE corrected). Both activation sites included about five voxels at FWE‐corrected level, and about 30 voxels at a more lenient threshold of *p* < 0.001 uncorrected used for illustrative purposes (Figure 5).

**Table 2:** MNI Coordinates (*x*,* y*,* z*), *Z* score of local maxima and cluster size (FWE *p* < 0.05 corrected and uncorrected *p* < 0.001 levels) of action discrimination‐specific sites in experiments 1 and 2

Location	Experiment 1				Experiment 2			
	*x*,* y*,* z*	*Z* score	Size		*x*,* y*,* z*	*Z* score	Size	
			FWE	Uncorr			FWE	Uncorr
L phAIP	−50 −38 44	5.7	7	31	−42 −46 40	5.7	5	29
R phAIP	42 −38 48	5.7	4	27	30 −50 42	5.8	6	22
L preCS					−40 −4 46	5.3	3	19

**Figure 6:**
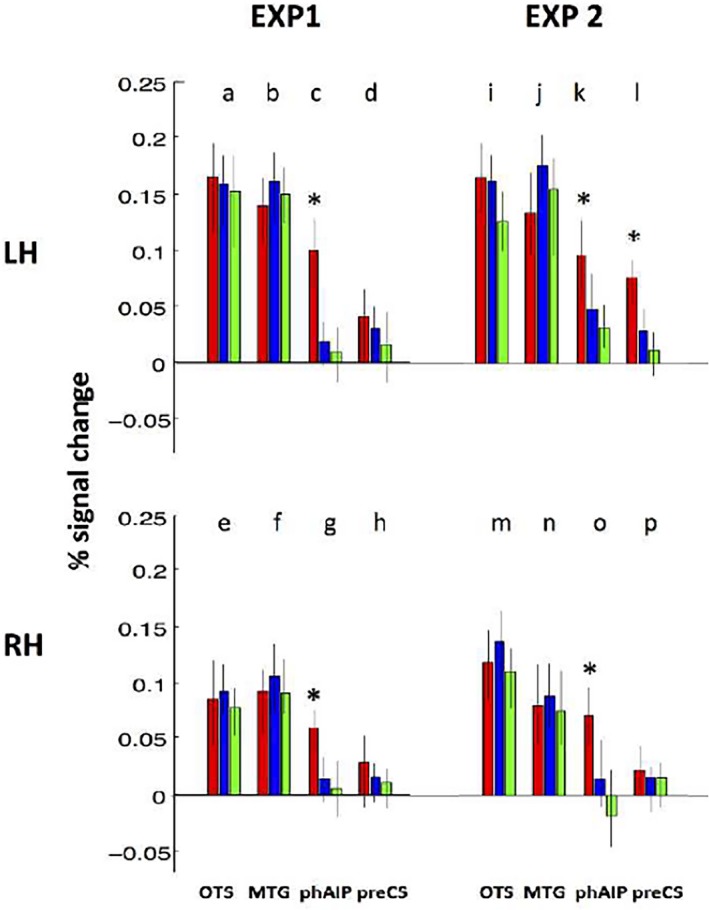
Activity profiles (red: action, blue: actor and green: colour discrimination) of the ROIs covering the action observation network (see Figure 3). Profiles of left (a–d, i–l) and right (e–h, m–p) hemispheres in experiments 1 (a–h), 2 (i–p). OTS, occipito‐temporal sulcus; MTG, middle temporal gyrus; phAIP, putative human AIP; preCS, precentral sulcus. Vertical bars: standard errors (*SE*s). See Tables 3 and 4 for significance of the three‐way and one‐way ANOVAs. Asterisks indicate that action condition is significantly more activated than the other conditions at *p* < 0.001, as shown by one‐way ANOVA

To further demonstrate the specificity of the phAIP sites, we performed a ROI analysis computing the percent signal changes in the three task conditions relative to active fixation (Figure 6) in sites defined a priori, MTG, OTS, phAIP and PreCS (Figure 3), taken from Ferri et al. (2015). A three‐way ANOVA with factors, *ROI, hemisphere* and *task*, yielded significant main effects of all factors and more importantly a significant interaction between ROI and Task (*F*_6,138_ = 3.5, *p* < 0.005, Table 3). Post hoc analysis indicated that activity was significantly stronger for action than the other discriminations in left phAIP (one‐way ANOVA *F*_2,48 _= 9.4, *p* < 0.001, Table 4) at corrected level and right phAIP at uncorrected level (*F*_2,48_ = 4.5, *p* < 0.01**)**, highlighting that featural attention to OMAs increases the activity of the parietal regions of the AON. The dorsal premotor PreCS ROI shown in Figure 6 is identical to that activated by observing manipulation in Ferri et al. (2015), the study from which the videos used in experiment 2 were also taken. As a control, we also applied the one‐way ANOVA to the ventral premotor ROI (vPM) that is activated by observing manipulation videos without any reaching component (Corbo et al., 2016). Again the ANOVA was not significant (*F*_6,128_ = 0.05, *p* > 0.9; Figure S2).

**Table 3:** Three‐way ANOVA results of univariate ROI analysis

	Main effects			Interactions			
	HEMI	ROI	COND	HEMI *ROI	HEMI* TASK	ROI* TASK	ROI*HEMI*TASK
Exp 1	***F***_**6,128**_ ** = 3.4** ***p*** ** < 0.05**	***F***_**6,128**_ ** = 2.7** ***p*** ** < 0.05**	***F***_**6,128**_ ** = 2.9** ***p*** ** < 0.05**	*F*_6,128_ = 1.5 *p* < 0.1	*F*_6,128_ = 1.7 *p* < 0.09	***F***_**6,128**_ ** = 3.9** ***p*** ** < 0.005**	*F*_6,128_ = 1.5 *p* < 0.2
Exp 2	***F***_**6,108**_ ** = 2.3** ***p*** ** < 0.05**	***F***_**6,108**_ ** = 2.4** ***p*** ** < 0.05**	***F***_**6,108**_ ** = 3.9** ***p*** ** < 0.04**	*F*_6,108_ = 1.6 *p* < 0.8	*F*_6,108_ = 0.5 *p* < 0.5	***F***_**6,108**_ ** = 3.9** ***p*** ** < 0.005**	*F*_6,108_ = 1.7 *p* < 0.16

Note

Bold indicates significant *F* value.

**Table 4:** One‐way ANOVA results of univariate ROI analysis

	OTS		MTG		phAIP		preCS	
	lh	rh	lh	rh	lh	rh	lh	rh
Exp 1	*F*_2,48_ = 0.07 *p* > 0.8	*F*_2,48_ = 0.08 *p* > 0.8	*F*_2,48_ = 0.09 *p* > 0.8	*F*_2,48_ = 0.1 *p* > 0.8	***F***_**2,48**_ ** =** **4.5** ***p*** ** < 0.001**	***F***_**2,48 **_ **= 5.3** ***p*** ** < 0.001**	*F*_2,48 _= 2.7 *p* > 0.07	*F*_2,48 _= 2.8 *p* > 0.07
Exp 2	*F*_2,36 _= 0.1 *p* > 0.9	*F*_2,36 _= 0.08 *p* > 0.9	*F*_2,36 _= 0.09 *p* > 0.9	*F*_2,36 _= 0.1 *p* > 0.8	***F***_**2,36 **_ **= 8.1** ***p*** ** < 0.001**	***F***_**2,36 **_ **= 9.2** ***p*** ** < 0.001**	***F***_**2,36 **_ **= 8.3** ***p*** ** > 0.001**	*F*_2,36 _= 2.8 *p* > 0.06

Notes

Threshold corrected for multiple comparison: α = 0.006; Bold indicates significant results.

Thus, fMRI experiment 1 confirms our prediction of a bilateral activation in phAIP when subjects discriminated between OMA exemplars relative to the two other discrimination tasks. Neither the whole‐brain nor the ROI approach, revealed any contribution from the other two levels. While the occipito‐temporal ROIs were active but not task‐specific, the premotor regions showed little or no activity.

### 3.2 fMRI Experiment 2

In the second fMRI experiment, we made the action discrimination task slightly more complex in the sense that subjects had to distinguish between the actor grasping an object with the right hand alone and pushing it bimanually.

All 19 successfully scanned subjects fixated well during scanning (average: 10.2 saccades per min, *SD* = 2.9), with no significant differences across conditions in the total number of saccades (ANOVA *F*_3,16_ = 0.3, *p* > 0.9). Mean response accuracy was again high in all three tasks: action = 95.5% (*SD* 1.8%), actor = 94.7% (*SD* 2.1%), colour 95.1% (*SD* 1.4%), but here, too, mean reaction times were longer for action discrimination (0.8 s, *SD* 0.65 s) than for actor (0.51 s, *SD* 0.23) or colour discrimination (0.49 s, *SD* 0.44 s).

The contrast ‘action discrimination versus active fixation’ showed that all three levels of the AON were activated in this experiment, in addition to some of the retinotopic regions (Figure 4). Again the activation pattern was biased towards the left hemisphere.

The conjunction of the contrasts comparing OMA discrimination condition with the remaining two discriminations yielded three specific regions for action (red in Figure 5). Bilateral phAIP (left: −42 −46 40, z = 5.7, *p* < 0.05 FWE corrected; right: 30 −50 42, *z* = 5.8, *p* < 0.03 FWE corrected) and left premotor cortex (−40 −4 46, *z* = 5.3, *p* < 0.05 FWE corrected) were more strongly activated by the action discrimination task than the other two discriminations (Figure 5c/d). Premotor activation in the right hemisphere did not reach FWE‐corrected level. Activation sites were again small (Table 2) and for illustrative purposes (Figure 5) they are plotted at a lower threshold of *p* < 0.001 uncorrected yielding about 20–25 voxels from each site. Notice that the left precentral activation was also observed in the general activation map, indicating that its activation in the second experiment did not reflect differences in control conditions but a difference related to the experimental condition (action discrimination).

Again, the ROI analysis (Figure 6e–h) confirmed the whole‐brain results. In the three‐way ANOVA, all main effects reached significance, as well as the interaction *ROI *×* Task*: (*F*_6,108_ = 3.9, *p* < 0.005, Table 3). Post hoc analysis (Table 4) indicated that activity is significantly stronger (at corrected level) for than the other discriminations in left phAIP (one‐way ANOVA *F*_2,36_ = 6.4, *p* < 0.001**)**, right phAIP (*F*_2,36_ = 7.1, *p* < 0.001) and left preCS (*F*_2,36_ = 6.6, *p* < 0.001). Thus the ROI analysis confirms a noteworthy variation with regard to experiment 1: the activation of left preCS in the action discrimination task.

The second fMRI experiment thus also bears out our predictions. The two experiments together clearly indicate the specific involvement of phAIP in observed manipulative action discrimination. While the occipito‐temporal level of the AON was again active in a nontask‐dependent manner, this time at least left premotor cortex also demonstrated task specificity.

## 4 DISCUSSION

Our results clearly favour the first of the two alternatives outlined in the introduction: OMA discrimination engages only the parietal AON level versus it engages both parietal and other levels. Indeed, bilateral activations of phAIP were observed in both experiments 1 and 2, in addition to a left premotor activation in experiment 2.

### 4.1 Comparison with previous studies

The longer reaction times in action compared to the two other discriminations, observed in both experiments, may partially reflect the difference in time course between the features used as discriminanda. Indeed the colour of the object and gender of the actor are available immediately from the first frame, whereas the action information requires a minimum of frames to be presented, as demonstrated by the duration thresholds of action discrimination (Platonov & Orban, 2016). Additionally, longer reaction times may also reflect difference in the time needed for processing the features. Ibos and Freedman (2014) reported that neurons in lateral intraparietal sulcus (LIP), an area adjacent to AIP in the monkey intraparietal sulcus, respond to coloured stimuli with latencies close to 50 ms, while AIP neurons respond to OMAs with latencies closer to 100 ms (Lanzillotto et al., 2019); Also the readout of the neuronal population activity seems faster in LIP for colour than in AIP for OMAs: the ROC of the LIP population reached its maximum in about 100 ms (Freedman & Ibos, 2018), while maximum decoding accuracy for OMAs was reached around 280 ms after video onset. Finally, additional delays may be introduced into the decision processes as evidence may accumulate at a slower rate for one of the two features for reasons completely unrelated to difficulty, such as strength of anatomical connections.

The alternative interpretation of the reaction time differences, a difference in task difficulty is less likely. In this alternative view, the phAIP activation would be interpreted as reflecting the increased level of overall attention rather than attention to different features. The factor difficulty in action observation has been explicitly manipulated by Lingnau and Petris (2013), who presented point‐light actions blended with different levels of dynamic noise. They reported significant main effects of noise, and hence difficulty, at the occipito‐temporal level of the AON, as well as in right lateral PFC. The noise main effect did not reach significance in either left or right IPL ROI, close to phAIP ROIs (their Table 2). Some studies have reported domain general effects of difficulty in parietal regions (Fedorenko, Duncan, & Kanwisher, 2013); however, the left IPS region (−37, −56, 41) reflecting general difficulty in that study was located more than 15 mm caudal to the phAIP (center −40, −40, 40). A similar discrepancy in location appears in the parietal region reported in a study manipulating difficulty in a speed discrimination task (Sunaert, Van Hecke, Marchal, & Orban, 2000). Thus the phAIP activation in experiments 1 and 2 is unlikely to reflect the difficulty of the action task compared to the other two discriminations, but rather attention to the observed‐action feature.

### 4.2 First alternative: only phAIP activation in OMA discrimination

The two fMRI experiments established the specific bilateral activation of phAIP in action discrimination compared to actor and colour discrimination, clearly supporting the first alternative. Even if the individual activation sites were small at the corrected level, they were bilateral and repeated in the two experiments, hence reinforcing each other. The activation sites in the two experiments did not overlap, with the site in experiment 2 being located more caudally in both left and right phAIP ellipses than that in experiment 1. This slight difference in location may reflect differences in subjects, exemplars discriminated, degree of generalization or the effector. Similar small shifts of the activations within phAIP were observed when comparing previous passive fMRI experiments (Abdollahi et al., 2013; Corbo & Orban, 2017; Ferri et al., 2015; Jastorff et al., 2010).

The bilateral activation of phAIP in experiments 1 and 2 are likely to reflect featural attention (Treue & Martinez Trujillo, 1999), as described in experiments using similar designs by Cant and Goodale (2007), Peuskens et al. (2004) and Chiu et al. (2011). Attention to one of the features present in a sensory stimulus enhances the activity of neurons selective for this feature (Martinez‐Trujilo and Treue, 2004), an effect that can be captured with univariate analysis of fMRI (Stoppel et al., 2011). Such featural attention effects are particularly strong at the parietal level as recently reviewed by Freedman and Ibos (2018), who reported that many LIP neuron showed little selectivity in passive conditions but became selective when the monkey performed the double conjunction task. The reports of OMAselectivity of AIP and phAIP neurons (Lanzillotto et al., 2019; Orban et al., 2016) suggest that neuronal tuning is similarly enhanced in phAIP when subject perform the action discrimination task, providing a mechanistic interpretation of the increased activation documented in phAIP.

### 4.3 First alternative: little task‐specific activation in PM and none in OTC

Our results did not provide much support for the second alternative: OMA discrimination engages both parietal and other AON levels. The absence of PCS activation in experiment 1 cannot be attributed to a statistical lack of power as this study included five more subjects than experiment 2, where the PCS activation was present. The absence of a featural attention effect for manipulative actions in premotor cortex may suggest a more limited role for the premotor level for perceiving others actions, which is consistent with a number of studies (Buxbaum & Kalenine, 2010; Caramazza, Anzellotti, Strnad, & Lingnau, 2014; Negri et al., 2007; Rogalsky et al., 2013; Stasenko, Garcea, & Mahon, 2013).

Yet premotor activation was observed in experiment 2, and the psychophysical control experiment using brief static‐probe stimuli makes it unlikely that this premotor activation reflects the static cues available at the start of the OA. The PCS activation in experiment 2, in which the two OMAS also differed with respect to the effector is consistent with our view (Orban, 2018) that while action identity is processed at the parietal level, essential, more detailed aspects of the observed action, such as the effector used and the kinematics (relative to the external world) are processed at the premotor level. Tracer studies in monkeys (Borra, Gerbella, Rozzi, & Luppino, 2011; Gerbella, Borra, Tonelli, Rozzi, & Luppino, 2013) have demonstrated that AIP, receives strong prefrontal input from prefrontal areas 12r and 46v. These inputs may, amongst others, control the flow of OMA‐related signals from AIP to premotor cortex. If similar connections are present in humans, they may indeed explain how for some instances of action observation premotor cortex can be recruited, in addition to phAIP.

The lack of a featural attention effect for manipulative actions in LOTC provides little support for the sort of major participation of lateral occipito‐temporal cortex (LOTC) in action observation proposed by other investigators (Gallivan & Culham, 2015; Kable & Chatterjee, 2006; Lingnau & Downing, 2015; Tucciarelli, Turella, Oosterhof, Weisz, & Lingnau, 2015; Valyear & Culham, 2010; Wurm, Caramazza, & Lingnau, 2017; Wurm & Lingnau, 2015). Although ceiling effects cannot be ruled out entirely, they are unlikely for the right hemisphere, in which the activation of the LOTC ROIs reached only 60% of the level in the left hemisphere. According to Lingnau and Downing (2015) ‘a mosaic of focal, but partially overlapping, selective regions in LOTC represents specific information – about the shape of bodies, patterns of motion, affordances of tools, etc. – that forms the components of action representations, and diffuse patterns of activity across LOTC integrate these multiple local representations’. They argue that ‘such representations are suited, but not limited, to encode the means (e.g. kinematics, hand posture, position of the hand with respect to the object) by which actions are carried out” and add that they ‘draw together actions that may have different local kinematic or perceptual features, but that share the aim to change the state of the world in a particular way, irrespective of how exactly this change of state is achieved’. This description of what can be signalled by LOTC representations is very close to our definition of the identity of an observed action: the goal of another's action (in relation to the world) and how the actor's movements allow him to reach the goal (Orban, 2018). Yet, the views of Lingnau and Downing were based in part on the results of MVPA analysis of MR activity to identify areas that can discriminate between different observed actions (Oosterhof et al., 2012), a strategy frequently used in subsequent studies (Hafri, Trueswell, & Epstein, 2017; Wurm, Ariani, Greenlee, & Lingnau, 2016; Wurm & Lingnau, 2015; Wurm et al., 2017). While we agree with the notion of the representation of action components in LOTC, we suggest that the single neuron data indicate that the identity of observed actions is represented at the parietal level. In our view, the low levels of accuracy generally reached by MVPA on fMRI (less than 60%, where chance is at 50%) data blurs the difference in action representation at the two levels, although some small differences have been reported (Wurm & Lingnau, 2015; Wurm et al., 2016). In contrast, our univariate technique picks up the distinction in featural attention effects at the two levels, suggesting a difference in nature between the representations at these two levels.

### 4.4 A general PPC function for building sensory representations for decisions signaled by motor responses

The studies of Freedman and Ibos (2018) suggest that LIP is involved in building a sensory representation that allows subjects to decide between two alternatives (red and yellow dots moving in different directions) and signal their decision by a saccade. An inference that has been little appreciated is that this finding implies that representations for perception and for reporting perceptual events by a motor response, in discrimination and in many other tasks, are distinct, a conclusion also supported by an earlier colour‐discrimination imaging study (Claeys et al., 2004). Combining those LIP studies with the present work suggests that building sensory representations for deciding between alternative motor responses, whether saccades, button presses, or perhaps verbal responses, may be a general function of posterior parietal cortex (PPC) areas. This function differs from building the well‐established representations that underlie the sensorimotor transformations involved in planning actions (Andersen, Snyder, Bradley, & Xing, 1997). OMA‐selective neurons, however, may contribute to both functions. Indeed, this study suggests that they contribute to building sensory representations to be used for motor reporting, while Lanzillotto et al. (2019) have suggested that they contribute to the planning of manipulative actions. Given the stereotypy of saccades this distinction may be less apparent for LIP, perhaps explaining the debate between intention and attention for this area (Colby & Goldberg, 1999; Freedman & Ibos, 2018; Snyder, Batista, & Andersen, 1997). That neurons selective for observed actions play a role in both functions is consistent with recent findings that observing actions of different classes drive those PPC regions that are involved in the sensorimotor transformation underlying the planning of actions of that class. (Abdollahi et al., 2014; Corbo & Orban, 2017).

### 4.5 Conclusions

Our study emphasizes the role of the parietal cortex in action observation, particularly in the representation of OA identity, which is encoded by OMA‐selective neurons in phAIP, and allows deciding between alternative observed manipulative actions.

## AUTHORS’ CONTRIBUTIONS

GAO and AP designed the study and wrote the manuscript; SF and AP collected the data; GAO, SF and AP analysed the results..

## CONFLICT OF INTEREST

The authors declare no conflict of interest financial or otherwise.
